# Novel methods for in vitro modeling of pancreatic cancer reveal important aspects for successful primary cell culture

**DOI:** 10.1186/s12885-020-06929-8

**Published:** 2020-05-13

**Authors:** L. Ehlen, J. Arndt, D. Treue, P. Bischoff, F. N. Loch, E. M. Hahn, K. Kotsch, F. Klauschen, K. Beyer, G. A. Margonis, M. E. Kreis, C. Kamphues

**Affiliations:** 1 Department of General, Visceral and Vascular Surgery, Charité - Universitätsmedizin Berlin, Berlin, Germany; 2 Institute of Pathology, Charité - Universitätsmedizin Berlin, Berlin, Germany; 3 Department of Surgery, Johns Hopkins University School of Medicine, Baltimore, USA

**Keywords:** Primary cell culture, Organoids, PDAC

## Abstract

**Background:**

Pancreatic cancer remains a fatal disease. Experimental systems are needed for personalized treatment strategies, drug testing and to further understand tumor biology. Cell cultures can serve as an excellent preclinical platform, but their generation remains challenging.

**Methods:**

Tumor cells from surgically removed pancreatic ductal adenocarcinoma (PDAC) specimens were cultured under novel protocols. Cellular growth and composition were analyzed and culture conditions were continuously optimized. Characterization of cell cultures and primary tumors was performed via hematoxylin and eosin (HE) and immunofluorescence (IF) staining.

**Results:**

Protocols for two- and three-dimensional PDAC primary cell cultures could successfully be established. Primary cell culture depended on dissociation techniques, growth factor supplementation and extracellular matrix components containing Matrigel being crucial for the transformation to three-dimensional PDAC organoids. The generated cultures showed to be highly resemblant to established PDAC primary cell cultures. HE and IF staining for cell culture and corresponding primary tumor characterization could successfully be performed.

**Conclusions:**

The work presented herein shows novel and effective methods to successfully establish primary PDAC cell cultures in a distinct time frame. Factors contributing to cell growth and differentiation could be identified with important implications for further primary cell culture protocols. The established protocols might serve as novel tools in personalized tumor therapy.

## Background

Pancreatic cancer is one of the leading causes for cancer related deaths worldwide [1]. Pancreatic ductal adenocarcinoma shows a dismal prognosis with a 5-year survival rate of 9% [2]. Surgery remains the only curative treatment option, but 80% of patients with PDAC are diagnosed in a locally advanced or metastatic tumor stage and are not eligible for surgery [3]. Despite intensive research efforts and advances in systemic therapies, the median survival for patients with metastatic PDAC remains less than a year [4]. As patients with PDAC show a highly heterogeneous response to chemotherapeutic agents, there is a necessity to develop personalized therapeutic strategies [5–7]. Adequate preclinical models are needed, taking into account, that resistance and response to cytotoxic therapies are affected by a complex interaction between mutational activity, intra- and intercellular signaling pathways and cellular tumor composition [8–11]. Research has been focused on cell lines for various experiments, but they do not reflect the in vivo situation [12–14]. It could be shown, that established pancreatic cancer cell lines display a completely different genetic structure than clinical samples of PDAC [15]. Primary cells from tumor tissue can display the same properties as the originating tumor and two- and three-dimensional primary cell culture models from PDAC proved to be an excellent platform to study tumor morphology and biology [16, 17]. However, efforts to develop culture methods in an effective, time sparing manner and recreating the in vivo conditions in an in vitro setting have been challenging [11, 16–18]. A multitude of factors are involved in primary cell culture models: Tissue digestion techniques, culture media, growth and differentiation factors as well as extracellular matrix components [8, 19, 20]. In the study presented herein, we established methods to create two- and three-dimensional primary cell cultures from surgically resected PDAC specimens. We focused on generating cell cultures recapitulating distinct features of the originating tumor and the evaluation of the aforementioned factors for successful cell growth and differentiation.

## Methods

### Human samples

After the informed patient’s consent, specimens of 14 patients with histologically proven PDAC who underwent a pancreaticoduodenectomy at the Department of General, Visceral and Vascular Surgery, Charité - Universitätsmedizin Berlin, Germany, were collected and a tissue specimen was mechanically extracted from the center of the suspected tumor. The tissue specimen was placed in a 50 ml tube containing 10 ml CMRL medium (Thermo Fisher) with penicillin/streptomycin (100 μg/ml, Biochrom) and amphotericin b (2,5 μg/ml, Biochrom), named Washing medium I (patient 01–11), or Advanced RPMI 1640 Medium (Thermo Fisher) with 100 μg/ml penicillin/streptomycin, named Washing medium II (patient 12–14) and was transferred on ice to the laboratory of the Department of General, Visceral and Vascular Surgery, Charité – Universitätsmedizin Berlin. The average time from surgical removal of the pancreatic tumor to the beginning of the tissue dissociation protocol in the surgical laboratory was 30 min. All tumors of the originating specimen were examined for their histopathological properties.

### Coating

Cell culture plates were coated with collagen (10 μg/cm^2^, Corning, incubated for 1 h, washed with Dulbecco’s phosphate-buffered saline (DPBS, Thermo Scientific) (patient 01–10), poly-l-lysine (100 μl/cm^2^, Sigma, incubated for 30 min, washed with DPBS) (patient 02–10), or left uncoated (patient 03–06; 08–14).

### Culture media

Pancreas I medium (patient 01–08) consisted of CMRL medium, penicillin/streptomycin (100 μg/ml), amphotericin b (2,5 μg/ml), insulin-transferrin-selenium (5 μg/ml/ 5 μg/ml/ 5 ng/ml, Sigma), nicotinamide (10 mM, Sigma), bovine serum albumin (BSA, 2 mg/ml), hydrocortisone (0,48 μg/ml, Stemcell) and human epidermal growth factor (hEGF, 20–50 ng/ml, Sigma). ROCK inhibitor Y-27632 2 HCI (10–20 μM, Selleckchem) and retinoic acid (200 nM, Sigma) were used for patient 03–08. For the generation of Serum I medium (patient 09–11), fetal bovine serum (FBS, 10%, Invitrogen) was added to Pancreas I medium. Organoid I medium (patient 12–13) comprised Advanced RPMI 1640 Medium, 2% FBS, HEPES buffer (10 mM, Thermo Fisher), l-glutamine (4 mM, Thermo Fisher), penicillin/streptomycin (100 μg/ml), amphotericin b (2,5 μg/ml), insulin-transferrin-selenium (5 μg/ml/ 5 μg/ml/ 5 ng/ml), nicotinamide (10 mM), B-27 (1%, Thermo Fisher), hydrocortisone (0,48 μg/ml), fibroblast growth factor 2 (FGF2, 5 ng/ml, Stemgent), and hEGF (50 ng/ml). The medium was stored at 4 °C and used for one week. ROCK inhibitor Y-27632 2 HCI (10 μM), platelet-derived growth factor (PDGF, 1 ng/ml, Stemcell), insulin-like growth factor 1 (IGF, 1 ng/ml, Stemcell), fibroblast growth factor 10 (FGF10, 10 ng/ml, Sigma), retinoic acid (200 nM) and ascorbic acid (20 μg/ml, Santa Cruz) were freshly added. Organoid II medium (patient 14) consisted of Advanced RPMI 1640 Medium, HEPES buffer (10 mM), l-glutamine (4 mM), penicillin/streptomycin (100 μg/ml), amphotericin b (2,5 μg/ml), insulin-transferrin-selenium (5 μg/ml/ 5 μg/ml/ 5 ng/ml), nicotinamide (10 mM), B-27 (1%) hydrocortisone (0,48 μg/ml), FGF2 (5 ng/ml), and hEGF (50 ng/ml). The medium was stored at 4 °C and used for one week. ROCK inhibitor Y-27632 2 HCI (10 μM), PDGF (1 ng/ml), IGF (1 ng/ml), A 83–01 (1 μM), FGF10 (100 ng/ml), retinoic acid (200 nM), ascorbic acid (20 μg/ml), r-spondin-1 (RSPO-1, 500 ng/ml, Peprotech), wnt-3a (100 ng/ml, R&D Systems), [Leu^15^]-gastrin I (10 nM, Sigma), noggin (100 ng/ml, Miltenyi) and n-acetyl-l-cysteine 1 mM (Sigma) were freshly added.

### Establishment of two-dimensional cell cultures and PDAC organoids

For an overview, see Table 2. Tissue culture was performed under sterile conditions using a laminar flow hood. Upon arrival at the laboratory, the removed PDAC specimen was placed in a 35 mm petri dish. A part of the tissue was frozen in liquid nitrogen, another part was fixed in 4% formalin for 24 h. All the following steps were performed on ice, with cold medium and cooled instruments. The largest part of the specimen used for cell culture was weighed and covered with Washing medium I or II. Tissue fragments were mechanically dissociated as small as possible with two sterile blades (patient 01–08; 10–13) or a sterile blade and a forceps (patient 14). Enzymatic digestion was performed with with a mix of collagenase XI (1 mg/ml, Sigma), DNAse I (4 μg/ml, Sigma) and trypsin (Thermo Scientific), 1 ml per 0,1 g tissue, incubation time was 30 min at 37 °C and horizontal rotation with 300 rpm. Digestion was stopped with Washing medium I, 2 ml EDTA (50 mM, Thermo Scientific) and BSA (40 μl/ml, Roth). Another centrifugation step was performed with 450 g at room temperature, following resuspension in Pancreas I medium. The suspension was rinsed through a 100 μm cell strainer (Corning) and seeded onto cell culture dishes (patient 1–2). Samples from patient 03–07 and 10 were digested with Enzyme mix I (collagenase/dispase (1 mg/ml, Roche) and 4 μg/ml DNAse I), 1 ml (patient 03) or 2 ml (patient 04–07; 10) per 0,1 g tissue and incubated at 37 °C and horizontal rotation with 300 rpm for one hour. After 30 min, trypsin/accutase (Thermo Scientific, 100 μl per ml enzyme mix, patient 06), trypsin (100 μl/ml Enyme Mix I, patient 06–07) or accutase (100 μl/ ml enzyme mix, patient 10) were added. With Washing medium I, the suspension was resuspended several times with a 14 g cannula placed on a 20 ml syringe. The cell suspension was then centrifuged with 200 g at 4 °C for ten minutes, the supernatant was removed and the cell pellet was resuspended in Pancreas I medium (patient 03–07) or Serum I medium (patient 10) and rinsed over a 40 μm cell strainer, transferred to a 15 ml falcon and centrifuged with 330 g at 4 °C for ten minutes. The supernatant was again removed, the cell pellet resuspended in Pancreas I medium (patient 03–07) or Serum I medium (patient 10) and seeded onto one well of a six well cell culture plate. Samples from patient 11 were digested in two steps with collagenase/dispase and accutase, 30 min at 37 °C each with two centrifugation steps at 440 g for 5 min at 4 °C. After mechanical dissocation of samples from patient 12–14, for 250 mg of tissue, 1 ml of a freshly prepared enzyme mix was added (enzyme mix was named Enzyme Mix II). The enzyme mix consisted of DNAse I (1,25 unit/ml, Sigma) hyaluronidase V (250 unit/ml, Sigma), dispase (0,15 unit/ml, Corning) and elastase (0,025 unit/ml, Sigma). Collagenase XI (0,5 mg, Sigma) was added separately. Half of the enzyme mix was placed on the PDAC tissue and mixed with a cut pipette tip. The tissue was digested for 30 min at 37 °C. After this first digestion, the tissue medium mix was rinsed over the petri dish several times with a cut pipette tip and then again chopped with a sterile blade and treated with a syringe stamp. For the second enzymatic digestion, the second half of the enzyme mix and collagenase XI was added and incubated for 45 min at 37 °C with horizontal rotation at 75 rpm. After incubation, the tissue medium mix was again rinsed over the petri dish and then transferred to a 100 μm cell strainer placed upon a 50 ml tube. 1 ml Washing medium II was rinsed over the cell strainer four times to incorporate as much of the digested tissue as possible. Cells in the flow through were counted in a counting chamber and then centrifuged at 440 g for 5 min at 25 °C. Tissue fragment culture comprised the preparation of 1–2 mm tumor fragments with a sterile blade and placing of three to six pieces onto one well of a six well cell culture dish (patients 03–09). Primary cell culture medium was added after the tissue fragments were adjacent to the cell culture dish. Tumor fragments from samples from patient 08 and 10 were placed together with enzymatically digested tissue in one cell culture dish. For thin layer Matrigel based cell cultures (patient 05–11), the enzymatically digested and resuspended cell pellet (patient 05–10: primary cell culture medium; patient 11: primary cell culture medium with 5% Matrigel) or the preparated tumor fragments (patient 05–09) were seeded onto Matrigel coated cell culture plates (50 μl/cm^2^, Corning, incubated for 30 min at 37 °C). For thick layer Matrigel based cell cultures (patient 05–07; 09), the cell suspension was resuspended in Matrigel (150 μl/cm^2^), seeded onto non coated cell culture plates and solidified at 37 °C for 30 min. On top assays with resuspension of cells in Organoid I medium with 33% Matrigel (total volume 12 well: 750 μl; chamber slide (Corning): 300 μl) were performed for patient 12 and 13. Organoid I medium (12 well: 2 ml; chamber slide: 750 μl) was added and cells were incubated at 37 °C. The protocol for patient 14 included placing 200 μl Matrigel in each well of a 12 well cell culture dish (24 well, 120 μl; chamber slide: 120 μl) and incubation for 30 min at 37 °C to solidify. When convex Matrigel formation was observed, 50–100 μl of Matrigel were rinsed around the edges of the cell culture plate as described previously [21]. Cells were resuspended in 100 μl (24 well: 60 μl; chamber slide: 60 μl) Organoid II medium. 100 μl Matrigel were added (24 well: 60 μl; chamber slide: 60 μl) and 200 μl (120 μl) of the cell-Matrigel suspension were placed on the Matrigel coated cell culture dishes and incubated for 30 min at 37 °C. For the first passage, a cell-Matrigel ratio of 1:3 was used and seeded onto coated cell culture plates. Within all described methods, medium was changed every three to four days and cell culture growth and organoid formation was observed daily under the light microscope (Eclipse TS 100, Nikon). Pictures were taken with inverted light microscopes (Eclipse TS 100, Nikon; Primovert with Axiocam 105 color camera, Zeiss).

### Cell and organoid splitting

For passaging of cell cultures, trypsin (patient 05), trypsin/accutase (patient 06) collagenase/dispase (patient 11) and dispase (patient 12–13) were added until the whole dish was covered, incubated at 37 °C and centrifuged. The supernatant was removed, the cell pellet resuspended and seeded onto cell culture dishes. For mechanical splitting (patient 14), medium was carefully removed when organoids comprised more than 75% of the volume of the cell culture dish, 1 ml (12 well) of 4 °C cold DPBS were added and the organoids within the Matrigel were incorporated with a 1000 μl pipette tip and placed in a 50 ml tube with 15 ml 4 °C cold DPBS. The cell culture dish was rinsed again with DPBS, the solution was incorporated and added to the 50 ml tube. The suspension was then centrifuged at 300 g at 4 °C for five minutes, the supernatant was removed until 4 ml were left or the surface of the Matrigel-cell suspension at the bottom of the tube was reached, another 10 ml 4 °C cold DPBS were added, mixed and centrifuged again at 300 g at 4 °C for five minutes. The supernatant was removed, the cells were resuspended in primary cell culture medium and Matrigel and placed on two wells of a Matrigel coated cell culture dish as described before.

### Paraffin sections, hematoxylin and eosin and immunofluorescence staining

After fixation in 4% formalin for 24 h, primary tumor tissue was processed for paraffin embedding with a standardized protocol. For HE and IF staining, 3–4 μm tissue sections were prepared, mounted on a superfrost microscope slide, placed in a drying cabinet (45–50 °C) for 24 h and then dried at 25 °C for one week. Deparaffinization was performed with a standardized protocol. Tissue sections were stained with hematoxylin for 30 s to one minute and washed with tap water for six minutes. Eosin staining was performed for five minutes. Sections were incubated with distilled water, ethanol and Roti®-Histol, mounted with Roti-Histokitt (Carl Roth) and dried under a laminar flow hood for one day. For antigen unmasking, microscope slides were placed into boiling 10 mM sodium citrate buffer (pH 6.0) for 60 min, cooled for 30 min, washed with DPBS once for one minute and twice for five minutes. Paraffin sections were permeabilized with 0,5% Triton X-100 (Sigma) diluted in DBPS for 10 min and then washed with DPBS for one minute and twice for five minutes. Sections were blocked for 60 min with DPBS, 5% normal goat serum (Cell Signaling) and 1% BSA at 25 °C (blocking solution). Paraffin sections were incubated with 50–100 μl of primary mouse monoclonal antibodies against e-cadherin (Cell Signaling, diluted 1:50), carbohydrate antigen 19–9 (CA 19–9, Thermo Scientific, 1:100) or cytokeratin 19 (CK19, Thermo Scientific, 1:100) and rabbit monoclonal antibodies against cellular tumor antigen p53 (p53, Cell Signaling, 1:50) and vimentin (Abcam, 1:200) in blocking solution overnight at 4 °C. Sections were washed with DPBS for one minute and three times for 15 min and then incubated for 60 min at 37 °C with 50–100 μl goat anti mouse Alexa Fluor 594 (Thermo Scientific, 1:250) and goat anti rabbit Alexa Fluor 488 (Abcam, 1:500) secondary antibodies and 4′,6-diamidino-2-phenylindole (DAPI, 1:10000) with 1% BSA. Sections were washed with PBS for one minute and three times for 15 min and cleansed with distilled water and embedded in antifade mountant (ProTaqs® Mount Fluor). Glass plates were placed into cell culture dishes and seeding of the digested cell suspension or tissue fragment was performed as described. Medium was removed and cells were fixed with paraformaldehyde (Electron Microscopy Sciences, 2–4%) or acetone-methanol for 5–30 min at 25 °C, permeabilized with triton X-100 (Sigma, 0,5%) and incubated with blocking solution containing 1% BSA and 5–10% normal goat serum or a combination with triton X-100 (0,1%) and tween20 (Promega, 0,1%) diluted in DPBS for 60 to 90 min. Primary cells were incubated with primary mouse monoclonal antibodies against CA 19–9 (1:250), CK19 (1:250), e-cadherin (1:30) and rabbit monoclonal antibodies against vimentin (1:250) and p53 (1:50) overnight at 25 °C in blocking solution. Cells were washed with DPBS three times, incubated for 30–120 min at 25 °C with 300 μl goat anti mouse (1:250) and goat anti rabbit (1:500) secondary antibodies and DAPI diluted 1:5000 in blocking solution and washed with DPBS three times. In chamber slides, medium was removed and primary cells containing Matrigel was washed with DPBS. Cells were fixed with 750 μl paraformaldehyde (2–4%) and glutaraldehyde (SERVA, 0,5%) diluted in DPBS for 30 min and then washed three times for ten minutes with glycine (TH.Geyer, 100 mM) diluted in DPBS. Permeabilization was performed with 750 μl triton X-100 (0,5%) for ten minutes. The organoid containing matrigel was washed with 750 μl tween 20 (Promega, 0,1%), diluted in DPBS three times for 10 min and incubated with 750 μl blocking solution containing 1% BSA and 10% normal goat serum or a combination with fab fragment goat anti-mouse (Jackson Immuno Research, 20 μg/ml), triton X-100 (0,1%) and tween 20 (0,1%) diluted in DPBS for 90 min. Another washing step with 750 μl tween 20 (0,1%) three times for 20 min with light horizontal rotation was performed. All steps were performed at 25 °C and between each step, Matrigel and organoid structure were controlled under the light microscope. Organoids were incubated with 300 μl of primary mouse monoclonal antibodies against CA 19–9 (1:50), CK19 (1:50), e-cadherin (1:30) and rabbit monoclonal antibodies against vimentin (1:100) and p53 (1:50) in blocking solution without fab fragments overnight at 25 °C. Chamber slides were washed with 750 μl tween (0,1%) four times for 20 min with light horizontal rotation and then incubated for 90 min at 25 °C with 300 μl goat anti mouse (1:250), goat anti rabbit (1:500) secondary antibodies and DAPI diluted 1:5000 in DPBS with 1% BSA, 10% goat serum, triton X-100 (0,1%) and tween 20 (0,1%) and washed four times with 750 μl DPBS. Embedding in mountfluor antifade mountant was performed. All immunofluorescence images were obtained with a laser scanning microscope (LSM 510 META, Zeiss). For an overview of antibodies and dilutions used see additional Table 1.

**Table 1:** Patient data

***patient***	***T***	***N***	***M***	***G***	***R***	***L***	***V***
01	3	1	0	2	0	0	0
02	3	0	0	2	1	0	0
03	1	1	0	2	0	0	0
04	2	1	0	2	0	1	1
05	3	1	0	3	0	1	1
06	3	1	0	2	0	1	1
07	2	1	0	3	0	0	0
08	2	0	0	2	0	0	0
09	2	1	0	3	0	1	1
10	3	1	0	2	0	1	0
11	3	1	0	2	0	1	1
12	3	2	0	2	0	1	1
13	3	1	0	2	1	0	0
14	2	1	1	3	1	0	0

Pathological status of patients with histologically confirmed PDAC included in the study. TNM classification system of the “International Union Against Cancer” was utilized to describe extension of disease

## Results

### Patient demographic and histopathologic analysis

PDAC specimens of 14 patients who underwent a pancreaticoduodenectomy at the Department of General, Visceral and Vascular Surgery, Charité - Universitätsmedizin Berlin, Germany, were immediately collected after surgery with the informed patient’s consent. All patients underwent a pylorus preserving procedure. Nine female and five male patients with a mean age of 74 years were included in the study and displayed a heterogenic extension of their disease, as it is summarized in Table 1. All tumors were identified as pancreatic ductal adenocarcinomas by histopathological examination.

### Establishment of two-dimensional cell cultures and PDAC organoids

**Fig. 1:**
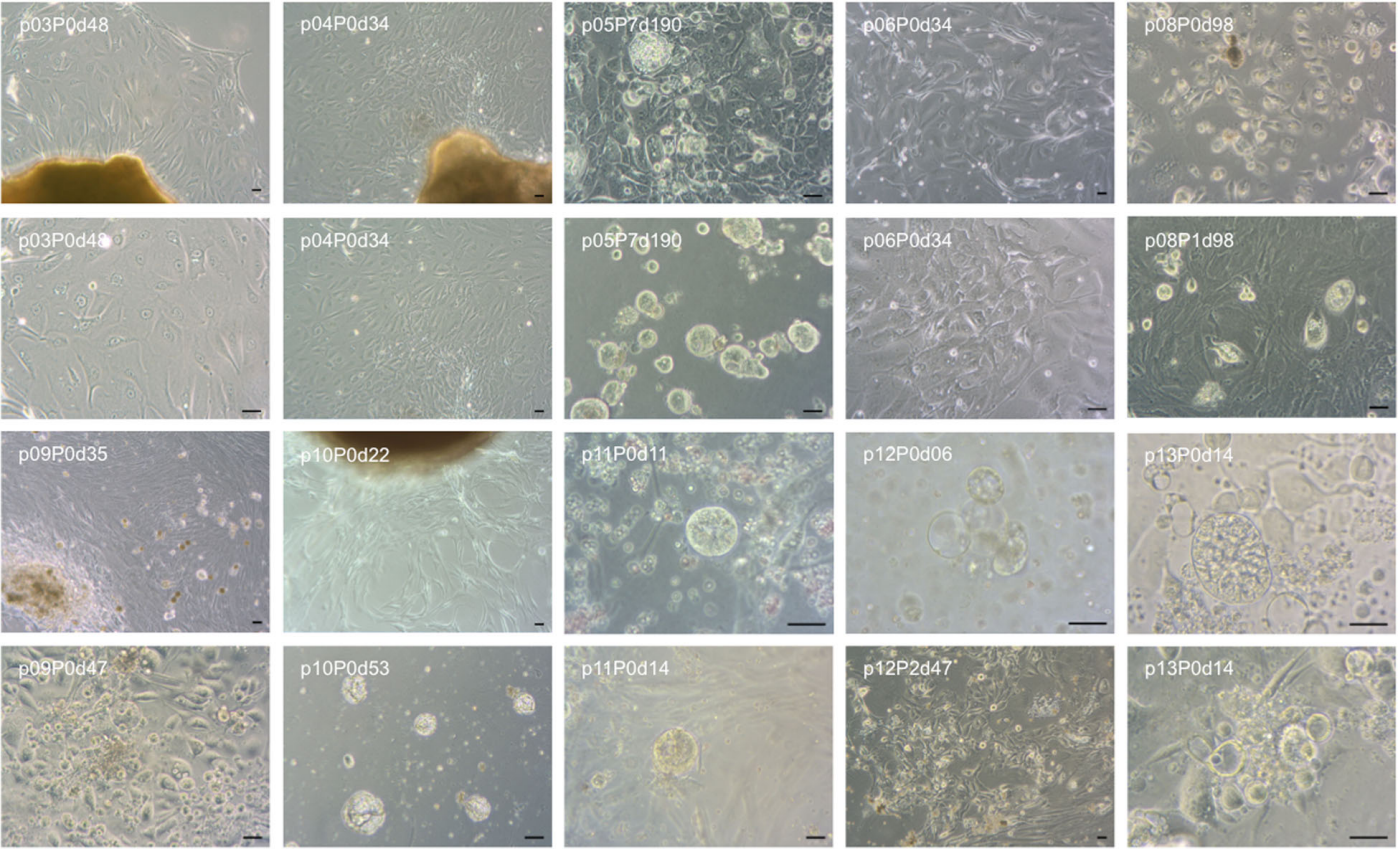
PDAC primary cell culture. Microscopic images of established primary cell cultures from patients 03–06 and 08–13 (representative images, *p* indicates patient number, P number of passages and d days after culture initiation). Scale bars, 20 μm

**Fig. 2:**
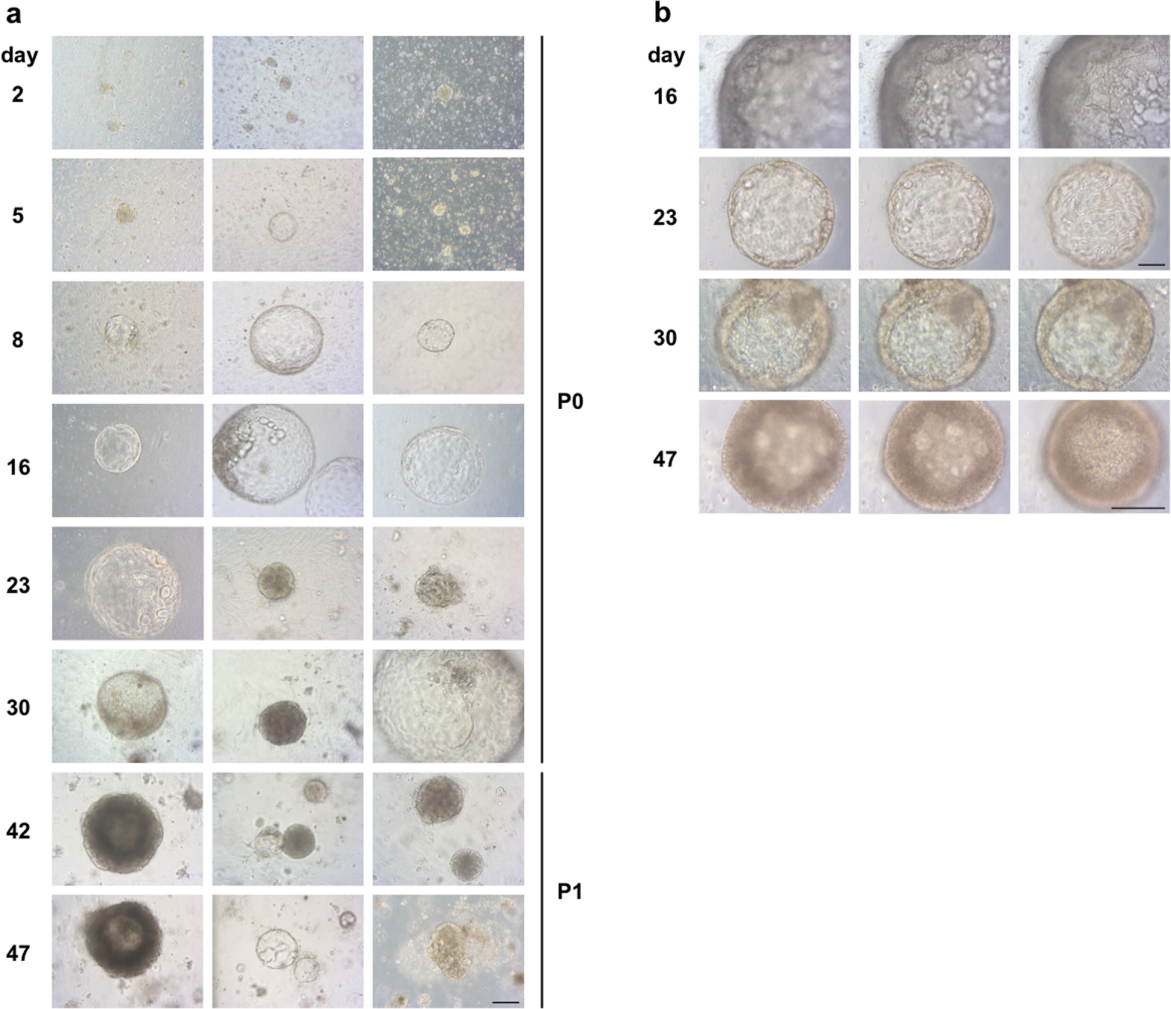
Establishment of PDAC organoid cultures. **a** Microscopic images of PDAC organoids from patient 14 (PDACp14cc), 2–47 days after culture initiation. Scale bars, 100 μm. **b** Different levels of three-dimensional organoids from PDACp14cc, 16–47 days after culture initiation. Scale bars, 100 μm

Primary cell cultures from tissue samples of 14 patients with PDAC could be initiated and 11 primary cell cultures could successfully be established (79%, named PDACpxxcc). Organoid formation could be observed in 6 (43%) initiated cell cultures. Multiple methods were performed and varied regarding coating of the cell culture plates, culture media, single cell and tissue fragment seeding, digestion enzymes and Matrigel composition. Cells could be propagated in culture for an average of 96 days (for an overview of performed culture techniques see Table 2). An enzymatic digestion protocol was performed with tissue samples from two patients without Matrigel (PDACp01/02 cc). No cellular growth could be observed (Table 2). Two cultures (PDACp03/04 cc) could be established via outgrowth from tissue samples adjacent to cell culture plates with a serum free cell culture medium based protocol (medium was named Pancreas I). Outgrowth from tissue samples could be observed for 144 (PDACp03cc) and 123 (PDACp04cc) days. Replacement of tissue samples onto novel cell culture dishes could be performed with similar outgrowth. Polygonal epithelial monolayers with almost rectangular shaped cells formed homogenous cobblestone-like patterns and were surrounded by elongated convoluted fibroblast-like cells. No difference in growth patterns between collagen or poly-l-lysine coated cell culture dishes could be observed, whereas a higher percentage of tissue fragments attached to collagen coated cell culture dishes (Fig. 1). Matrigel containing cell cultures with Pancreas I medium were performed. Primary cells were successfully established and propagated for 194 days and seven passages (PDACp05cc). Thin layer Matrigel culture with an overlay of a medium cell suspension was accomplished, as well as cell suspension in Matrigel and direct seeding on collagen coated cell culture dishes. After three days, outgrowth from tissue fragments embedded in Matrigel could be observed with polygonal cells in epithelial-like clusters. Enzyme mix I, consisting of collagenase/dispase and DNAse was used with an incubation time of one hour at 37 °C, after centrifugation, cells were seeded and formed clusters in Matrigel after seven days. Cobblestone-like cellular patterns with scattered round to oval cell islets with strong growth and small organoid-shaped formations could be observed (Fig. 1). Cell culture for PDACp06cc lasted 127 days with one successful passage of tissue fragments (Fig. 1). Enzymatic digestion with Enzyme mix I with and without trypsin/accutase showed a low yield, especially without trypsin/accutase. Another digestion step at day one was performed with Enzyme mix I and trypsin and cultures showed successful growth of elongated cells with rectangular shaped cell islets (Fig. 1). For PDACp07cc, no cellular growth could be spotted. With Pancreas I medium, primary cells from PDACp08cc lasted 113 days in culture, tissue fragments could be passaged once, cells could be passaged twice with a trypsin-based technique. Elongated fibroblasts could be noticed, especially when cells were attached to coated cell culture dishes. In Matrigel, patterns of scattered, oval cells and in progressing culture, polynuclear giant cells could be observed (Fig. 1). Tissue fragment culture was prepared with the PDACp09cc specimen with Serum I medium and lasted 81 days in culture. Outgrowth of convoluted and long fibroblasts from tissue fragments could be discovered, epithelial cells were scattered in Matrigel. Different serum containing media were tested. CMRL, DMEM and RPMI containing cultures with high serum concentration showed similar patterns of fibroblast growth with scattered and progressively reducing epithelial cells. The highest count of rectangular cells with epithelial morphology was observed with Pancreas I medium and Matrigel (Fig. 1). A combined approach of placing tissue fragments on a thin layer of Matrigel and an enzymatically (Enzyme Mix I + accutase) digested cell suspension was performed for PDACp10cc. Culture lasted 78 days, in Matrigel, scattered incomplete organoid formation and mixed cellular outgrowth with a high fibroblast count was observed (Fig. 1). With a two step enzymatic digestion protocol, thin layer Matrigel and 5% Matrigel containing cell culture medium, organoid growth for PDACp11cc could be noted. Cells which migrated to the bottom and attached to the cell culture dish formed mixed epithelial and fibroblast clusters, lasted 56 days in culture and could be passaged once (Fig. 1). A protocol with two enzymatic digestion steps using Enzyme Mix II, consisting of DNAse I, hyaluronidase V, dispase, elastase and Collagenase XI and two mechanical dissociation steps showed a high cellular yield. A concentration of around 1 × 10^6^ cells per cm^2^ cell culture dish could be achieved. A technique with resuspension in 33% Matrigel was performed and medium with a lower serum content consisting of 2% FBS, hGEF, b27, ROCK inhibitor, retinoic acid, FGF2, FGF10, PDGF and IGF as differentiation factors was used (Organoid I medium). Organoid outgrowth could be observed after six (PDACp12cc) and eight (PDACp13cc) days. Cells which migrated to the bottom of the cell culture dish showed a mixed epithelial and fibroblast morphology with progressing fibroblast growth. Organoids and cells lasted in culture for 51 days and two passages (PDACp12cc) or 39 days and two passages (PDACp13cc), respectively (Fig. 1). With the technique of combined two step mechanical and enzymatic digestion, a successful protocol for PDAC organoid culture could be established (Fig. 2). The implemented cell culture medium (Organoid II) was serum free with higher FGF10 concentrations (100 ng/ml). Noggin, rspo1, wnt3a and gastrin were added as differentiation and growth factors. Organoids lasted in cell culture up to 48 days and could successfully be passaged (Fig. 1). Matrigel concentrations were modified, for a well of a six well cell culture dish, coating with 200 μl cold Matrigel (24 well: 120 μl; chamber slide: 120 μl) was performed and incubated 30 min at 37 °C to reach optimal viscosity. The optimal viscosity for on top implacement of the cell-Matrigel suspension was reached when cells were resuspended in Organoid II medium with 50% Matrigel for at least 1 min (12 well: 200 μl; 24 well: 120 μl; chamber slide: 120 μl). With a cell-Matrigel ratio of 1:3, similar organoid growth patterns could be seen. After incubation for 30 min at 37 °C, Organoid II medium with 5% Matrigel was added and successful organoid growth could be observed (Fig. 2). Organoids formed three-dimensional structures with distinct central and peripheral cellular formations and resembled established PDAC organoid cultures [9, 19].

**Table 2:**
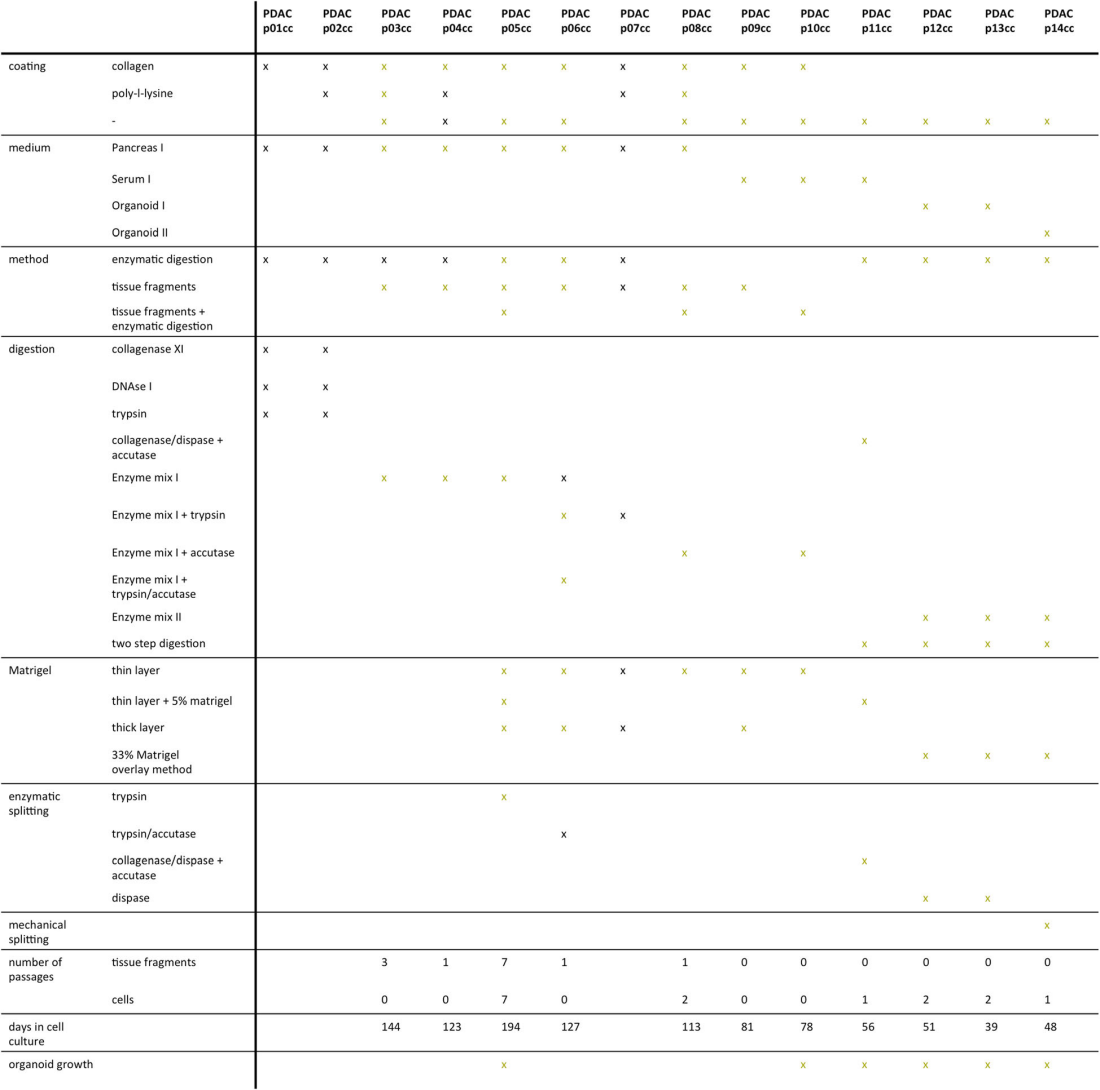
Overview of culture protocols

Culture protocols performed for patients included in the study. Yellow marked x indicates successful performance

### Characterization of primary tumors and cell cultures

**Fig. 3:**
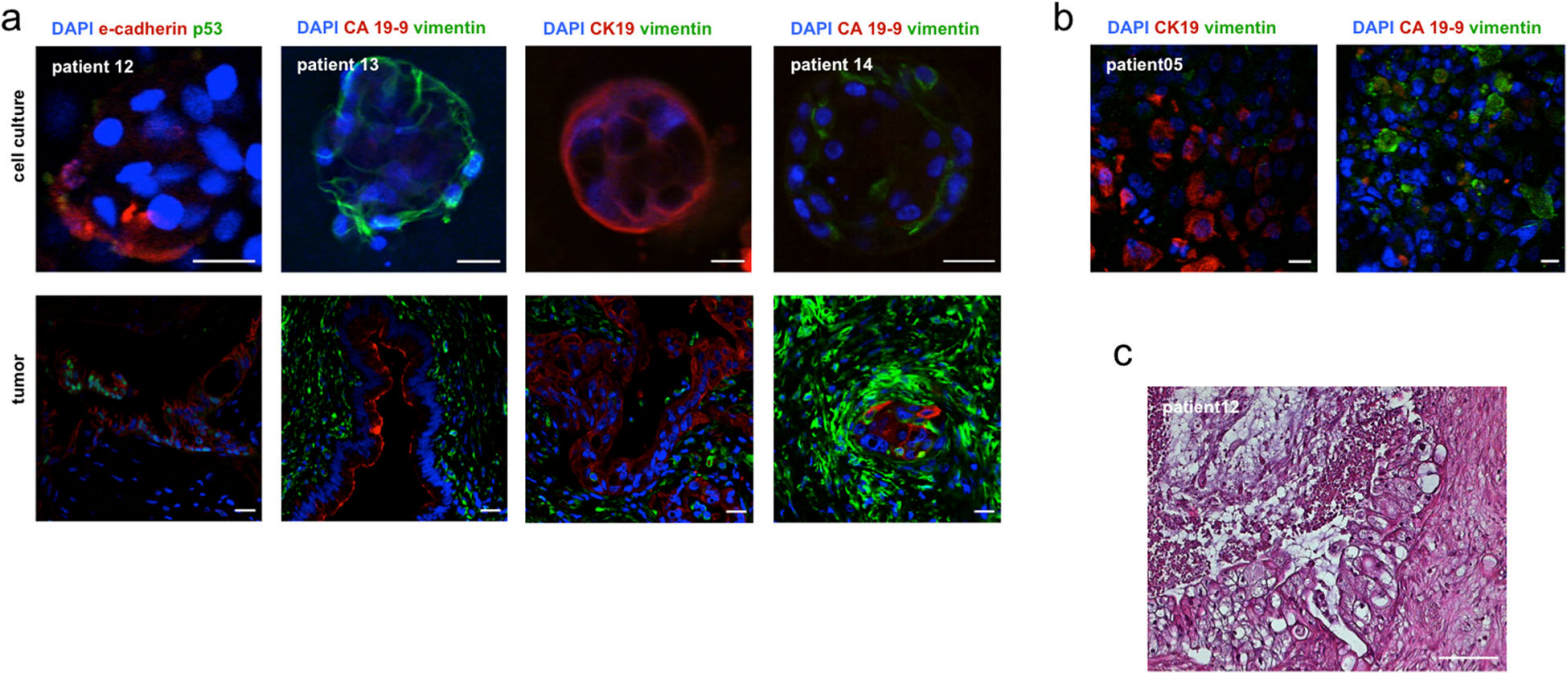
Immunofluorescence and hematoxylin and eosin staining. **a** Representative images of vimentin, CK19, CA 19–9, e-cadherin and p53 expression of primary cell cultures with organoid formation and corresponding tumors, indicated by IF staining. Scale bars, 20 μm. **b** IF staining of vimentin, CK19 and CA 19–9 expression of two dimensional primary cell cultures (representative images). Scale bars, 20 μm. **c** HE staining of primary tumors (representative image). Scale bars, 100 μm

HE staining was performed for tumor specimens of 3 patients (PDACp12–14 t). Paraffin sections revealed distinct pathological patterns. PDACp12t showed an epithelial desmoplastic morphology and ductal formations with luminal muzine retention, PDACp13t displayed characteristic tubulous epithelial neoplasia and PDACp14t exhibited cribriform and tubular ductal proliferation patterns with a marked desmoplastic stromal reaction as described in the clinical pathological report (Fig. 3c). IF staining of CA 19–9, CK19, vimentin, e-cadherin and p53 for primary cell cultures (PDACp03;05;9-11 cc), a comparative analysis of primary tumors from patients 12–14 and corresponding two- and three-dimensional primary cell cultures was performed. Expression of epithelial or PDAC specific markers could be detected in 87,5% of the stained primary cell cultures. Outgrowing cells from tissue fragments on poly-lysine coated glass plates from PDACp03cc showed vimentin expression with isolated CA 19–9 expressing cells after 89 days in culture. IF from PDACp05cc after 183 days revealed dissociated CA 19–9 and CK19 expression without formation of ductal structures with surrounding vimentin expressing fibroblasts. PDAC09cc displayed vimentin and isolated CK19 expressing cells after 55 days in culture. PDACp10cc showed vimentin expression, but no expression of epithelial markers or p53 was observed. PDACp11cc showed no p53 and CA 19–9 expression after one passage and 55 days in culture and displayed vimentin expressing fibroblasts, isolated CK19 expressing cells and islets with punctual e-cadherin expression (Fig. 3b, Table 3a). Intermediate to high vimentin expression of fibroblasts could be observed in all primary tumors, with scarce to intermediate vimentin expression of all correlating cell cultures. All primary tumors showed expression of the epithelial markers CK19 and e-cadherin and the PDAC specific marker CA 19–9. CA 19–9 expression was observed in glandular proliferates and could not be observed within poorly differentiated cell clusters. IF staining of the corresponding primary cell cultures revealed expression of CK19, e-cadherin or CA 19–9 in all patients. PDACp12cc showed scarce e-cadherin expression 14 and 34 days after culture initiation. PDACp13cc organoids displayed marked membranous expression of CK 19. E-cadherin expression could be observed in PDACp14cc. Simultaneous expression of CK19, e-cadherin and CA 19–9 could not be observed in primary cell cultures. IF staining of p53 was performed with one positive staining of p53 for the primary tumor sample of patient 12 without p53 expression of the corresponding cell culture (Fig. 3a, Table 3b).

**Table 3:**
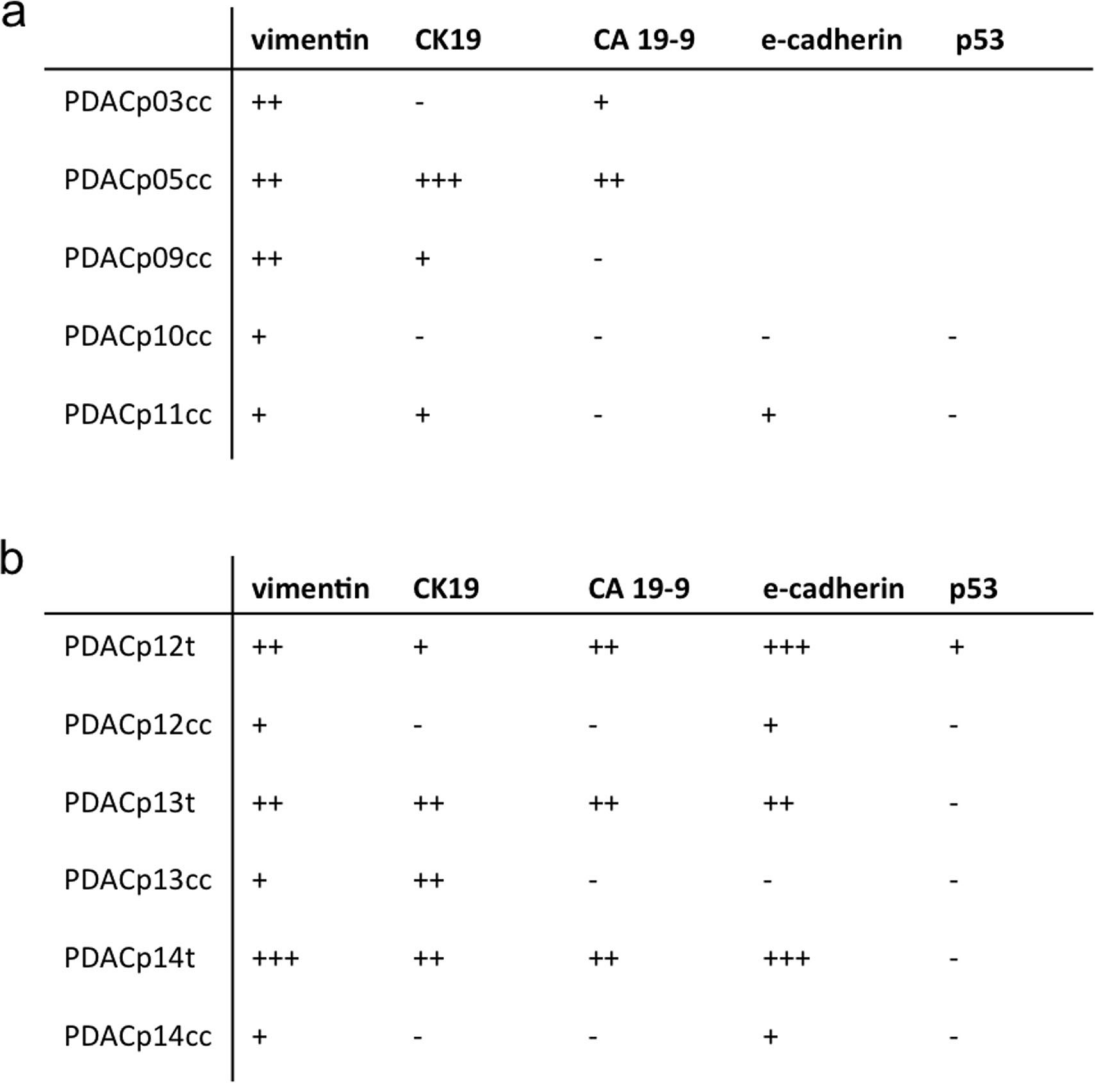
Expression patterns of immunofluorescence staining

Expression profiles of vimentin, CK 19, CA 19–9, e-cadherin and p53 from cell cultures (PDACpxxcc) (**a** + **b**) and primary tumors (PDACpxxt) (**b**), detected by IF staining. Expression patterns were defined as not present (−), scarce (+), intermediate (++) and high (+++). Blanc areas indicate non-performance due to limited tissue availability

## Discussion

Recapitulating cellular architecture by gentle yet efficient tissue dissociation techniques, recreating intercellular signaling and cellular differentiation by balanced growth factor supplementation and mimicking structures of extracellular matrices are important factors for successful cell culture [9]. PDAC shows a characteristic stromal microenvironment with cancer-associated fibroblasts, accessory cells and a dense meshwork of collagen, fibronectin, proteoglycans, hyaluronic acid, blood and lymphatic vessels [22, 23]. One of the hallmarks of PDAC primary cell culture is the dissociation of collagen type I and III predominant dense stromal structures to enable successful recapitulation of cellular structure in the in vitro setting [24]. Previous studies showed different, mostly mechanically and enzymatically combined approaches towards PDAC tissue dissociation with different collagenase types, dispase, DNAse and trypsin [8, 9, 19, 25, 26]. In our study, the most effective protocol comprised a two step approach by mincing with a combination of a sterile blade and forceps and crushing with a syringe stamp. The interaction between the two mechanical and two enzymatic digestion steps was crucial for optimal tissue penetration of digesting enzymes on the one hand and enzymatic tissue softening for optimal mechanical effects on the other hand. The most effective enzyme mix comprised collagenase XI, DNAse I and dispase II with efficient proteolytic activity towards interstromal cell connections, collagen type I and hyaluronidase V to hydrolyze glycosidic linkages in hyaluronic acid and elastase to address elastin fibers which are associated with lysyl oxidase-like 2 mediated tumor migration [27–31]. Cell culture media and added growth and differentiation factors play a pivotal role in successful cell culture initiation and should reflect in vivo conditions as realistic as possible [32]. Huch et al. described an organoid culture of pancreatic ducts from healthy mice and showed EGF, nicotinamide, wnt agonist r-spondin-1 and noggin, a protein involved in bone morphogenic protein (BMP) – 4 inhibition, to be essential for organoid formation and maintenance [33, 34]. Wnt signaling promotes epithelial-mesenchymal transition (EMT) in PDAC and the ligand wnt3a has been used in PDAC cell culture before [35]. In a study describing the establishment of PDAC organoids from surgically resected specimen with 80% efficacy, EGF, FGF10, noggin, r-spondin-1, wnt3a, A83–01, a TGF-β inhibitor, primocin and gastrin (a downstream target of the wnt pathway) were utilized [26]. Tsai et al. described a wnt pathway ligand and TGF- β requirement for successful organoid growth and used A83–01 and gastrin with a success rate of 76% [26]. Interestingly, in one study, retinoic acid, ROCK Inhibitor, insulin, hydrocortisone and DBZ, a notch pathway inhibitor were used, whereas no wnt3a and r-spondin are needed and noggin was employed only for stem cell derived pancreatic progenitor cultures [9]. Considering these heterogenous approaches and the complex alterations in signaling pathways through neoplastic progression, it is questionable how to translate insights from growth factor dependencies of stem cell and normal pancreatic tissue cultures into primary tumor cell culture, as even no correlation between organoid growth and specific additives is reported in literature [19]. In our study, BMP-4 and TGF-β inhibiting growth factors as well as r-spondin-1 and wnt pathway ligands played a pivotal role for organoid outgrowth but successful organoid outgrowth without dependency of the aforementioned growth factors could be observed as well. Here, fibroblast growth factor FGF2 and 10, hEGF, IGF and PDGF were used as PDAC shows an overexpression of their receptors, being associated with angiogenesis, desmoplastic reaction and tumor growth [36]. Varying effects of retinoic acid and ROCK inhibition are described in primary cell culture, from promotion to inhibition of cellular growth and differentiation [37, 38]. In our study, within two-dimensional cell culture, adding retinoic acid and ROCK inhibitor resulted in a boost of cellular growth. In our opinion, more studies are needed to evaluate the role of retinoic acid and ROCK inhibitor based culture media for PDAC cell culture.

Serum contents in cell culture media promote cellular growth, but with PDGF releasing effects, they can lead to fibroblast overgrowth [39]. In the work presented herein, culture with serum containing media was successful, but higher fibroblast growth in two-dimensional cell culture could be observed. With a serum free approach, enzymatic digestion protocols were not successful since cellular outgrowth could only be observed in adjacent tissue fragments with 100% efficacy. Ruckert et al. described an efficacy of 9,25% for the isolation of pancreatic cancer cell lines with a similar outgrowth method. Medium with a high serum content and without other growth factors was used, fibroblast overgrowth being the major problem during cell culture [16]. In the study presented by Kim et al., an efficacy of 7,4% was reached with a serum containing protocol and an intermittent fibroblast removal was performed to avoid overgrowth [40]. In our opinion, serum concentrations should be preferably low and specific factor containing approaches should be considered for successful outgrowth from tissue fragments. Besides the aforementioned culture media requirements, intercellular signaling especially from cell clusters, cellular density and differentiation signaling from extracellular matrix components seem to play a pivotal role in promoting cellular growth and differentiation [8, 17]. In the work presented herein, only a high cellular density with around 1 × 10^6^ cells per cm^2^ cell culture dish within enzymatic digestion protocols assured successful organoid outgrowth. To our knowledge, only one study evaluating initial cell seeding density exists so far, focusing on in vitro formation of pancreatic endocrine cell cultures from embryonic stem cells [41]. More studies are needed to evaluate standardized initial cell seeding counts for successful PDAC primary cell culture.

Matrigel provides structure and growth factors from the extracellular matrix and is an inevitable component for successful organoid formation [9]. In our study, outgrowth of two-dimensional cell clusters from tissue fragments was possible with low growth factor containing cell culture media and without Matrigel. The attachment of the fragments to cell culture dishes turned out to be very important. This could indicate, that the high cellular density of tissue fragment culture is sufficient to promote primary cell growth without highly differentiated cell culture media and without Matrigel. With enzymatic digestion protocols and single cell suspensions with less cellular density, only with Matrigel successful cellular growth could be observed, using extracellular matrix components not only for organoid culture but for monolayer cell cultures should be taken into account [42]. In the work presented herein, only the combination of Matrigel layers at the bottom of the cell culture dish with an overlay of a 1:1 cell suspension - Matrigel mixture, as well as an extension of the duration of Matrigel solidification processes at 37 °C, showed to be highly effective. Differing Matrigel composition could be observed. Based on our novel protocol, further studies should evaluate Matrigel solidification time and structure to ensure successful embedding of primary cells which was the hallmark of successful organoid formation.

Previous studies showed different approaches defining the “right” organoid morphology and characteristics, which resemble traits of the corresponding tumor [8, 9, 19, 25, 26, 43]. Baker et al. described organoid passaging for five or more times as sufficient organoid propagation, which was achieved in 66% of initiated cultures. No correlation between traits of the originating tumor and corresponding organoids has been made regarding cellular architecture or histopathological features [43]. Boj et al. described murine and human PDAC tumor models. Organoid morphology was shown, as well as HE staining of organoids and corresponding tumors. The analysis of resemblance was made through interpretation of cellular morphology of tumor organoids compared to organoids from non-malignant pancreatic tissue. Genetic properties of organoids were analyzed through targeted sequencing and showed heterogenous but present mutations typical for PDAC [19]. In another study, organoids of five patients were shown, presenting similar histological features and expression of the differentiation markers KRT19, GATA6 and SOX9 [9]. Regarding two-dimensional culture, successful establishment of 5 cell lines from 54 patients with PDAC, with successful initial outgrowth in 28 cases in one study and the establishment of six cell lines from 81 patients with PDAC were described. Comparative immunohistochemical analysis for CK8/18, CDH, ezrin, p53, vimentin, SMAD4, KRAS, CK19, p53 and DPC4 were performed, expression patterns were divided into three levels with varying accordance with the deriving tumor [16, 40].

Within the not yet standardized comparability assays regarding the in vivo situation, in the work presented herein, we provide a sufficient characterization of the generated cell cultures, taking into account that this study does not provide transcriptomic data as it could be described before [9, 19, 43]. The morphology of the established cell cultures was microscopically analyzed and showed similar cellular patterns as described PDAC primary cell cultures [9, 40]. For further characterization, IF staining of primary cell cultures and HE and IF staining of corresponding primary tumors could be performed with distinct expression patterns vimentin, CA 19–9, CK19 and e-cadherin. Nevertheless, weaker expression profiles of the stromal, epithelial and PDAC markers could be observed as the high cellular density and intercellular connections of primary tumors were not in total recapitulated in the generated primary cell cultures. In our opinion, further studies are needed to evaluate the efficacy of tissue culture characterization techniques regarding the predictability for successful application of the generated cell cultures in personalized medicine [8, 44].

## Conclusions

Whereas the majority of the previous studies developing primary cell cultures from PDAC used stem cell techniques or pancreatic explants from mice, to our knowledge, only three methods for the culture of two-dimensional primary cultures and only three methods for the culture of organoids derived from surgically resected PDAC specimens have previously been described [9, 16, 20, 26, 40, 43]. In our study, a comparatively high percentage of 79% initiated primary cell cultures from 14 patients with PDAC could successfully be established. The established cell cultures showed marked propagation capacities with an average of 96 days. Two non Matrigel dependent and 9 Matrigel dependent cultures with two-dimensional cellular cluster and 5 partial and one complete organoid formations could be generated. Tissue digestion methods, media supplements, cellular densitiy and intercellular signaling influenced the generated primary cell cultures. A profound analysis for the molecular basis of methodical success was made. Organoid formation showed to be highly dependent on viscosity and concentration of Matrigel. Indicated by organoid morphology, our results show that the developed organoids resemble traits of established organoid cultures [9, 19]. Primary cells were kept in culture for 1 or 2 passages as the aim of this study was not to create permanent cell lines, but passaging up to 7 passages was possible, proving the durability of the generated cell cultures. We present a valid protocol for HE and IF staining to characterize originating tumor specimen and primary cell cultures. Expression of epithelial or PDAC specific markers could be observed in 87,5% of the stained primary cell cultures, conserving tumor specific cellular composition.

## Supplementary information

**Additional file 1: Table S1.** Overview of antibodies and associated dilutions.

## Abbreviations

PDAC: Pancreatic ductal adenocarcinoma
HE: Hematoxylin and eosin
IF: Immunofluorescence
DPBS: Dulbecco’s phosphate-buffered saline
BSA: Bovine serum albumin
hEGF: Human epidermal growth factor
FBS: Fetal bovine serum
FGF: Fibroblast growth factor
PDGF: Platelet-derived growth factor
CA 19–9: Carbohydrate antigen 19–9
CK19: Cytokeratin 19
p53: Cellular tumor antigen p53
DAPI: 4′,6-diamidino-2-phenylindole
