# Supplementary figures and images for: Novel methods for in vitro modeling of pancreatic cancer reveal important aspects for successful primary cell culture
Source: BMC Cancer. 2020 May 13;20:417. doi: 10.1186/s12885-020-06929-8 (PMC7222463; doi:10.1186/s12885-020-06929-8)

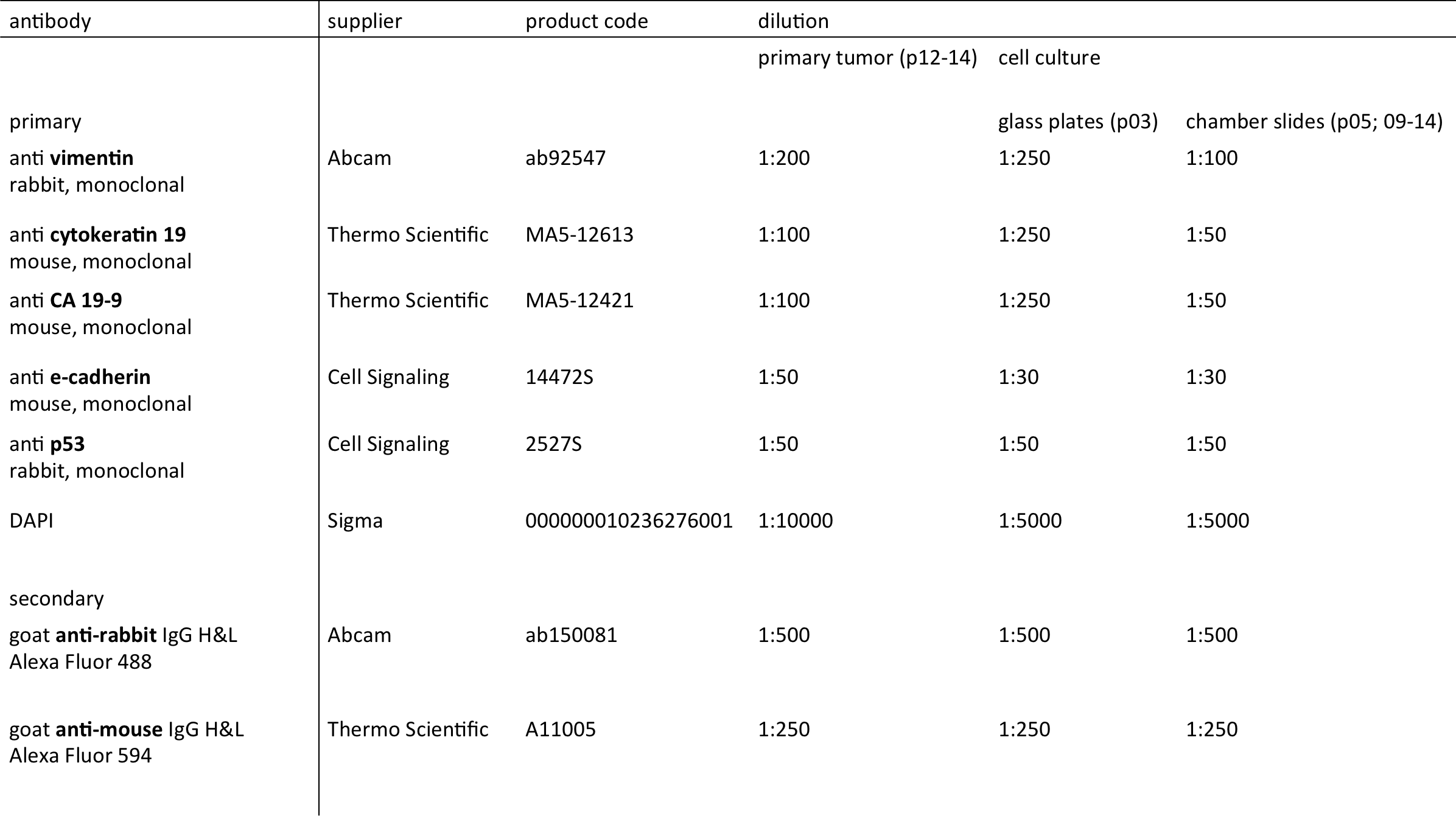

Supplement: Supplementary file 1 — Additional file 1: Table S1. Overview of antibodies and associated dilutions. [file 12885_2020_6929_MOESM1_ESM.png]
